# One health investigation following a cluster of Crimean–Congo haemorrhagic fever, North Macedonia, July to November 2023

**DOI:** 10.2807/1560-7917.ES.2025.30.4.2400286

**Published:** 2025-01-30

**Authors:** Dejan Jakimovski, Pavle Banović, Katerina Spasovska, Goran Rangelov, Marija Cvetanovska, Fadil Cana, Verica Simin, Ivana Bogdan, Dragana Mijatović, Aleksandar Cvetkovikj, Igor Djadjovski, Iva Christova, Eleftherios Meletis, Polychronis Kostoulas, Brigitta Zana, Zsófia Lanszki, Tamás Görföl, Zsófia Tauber, Gabor Kemenesi

**Affiliations:** 1 Faculty of Medicine, Ss. Cyril and Methodius University in Skopje, Skopje, North Macedonia; 2 University Clinic for Infectious Diseases and Febrile Conditions, Skopje, North Macedonia; 3 Clinical medicine Task Force, Balkan Association for Vector-Borne Diseases, Novi Sad, Serbia; 4 Department of Microbiology with Parasitology and Immunology, Faculty of Medicine in Novi Sad, University of Novi Sad, Novi Sad, Serbia; 5 Diagnostics and Laboratory research Task Force, Balkan Association for Vector-Borne Diseases, Novi Sad, Serbia; 6 Clinic for Lyme Borreliosis and Other Tick-Borne Diseases, Pasteur Institute Novi Sad, Novi Sad, Serbia; 7 Department for Microbiological and Other Diagnostics, Pasteur Institute Novi Sad, Novi Sad, Serbia; 8 Department for Research & Monitoring of Rabies & Other Zoonoses, Pasteur Institute Novi Sad, Novi Sad, Serbia; 9 Department of Parasitology and Parasitic Diseases, Faculty of Veterinary Medicine-Skopje, Ss. Cyril and Methodius University in Skopje, Skopje, North Macedonia; 10 Farm Animal Health Department, Faculty of Veterinary Medicine-Skopje, Ss. Cyril and Methodius University in Skopje, Skopje, North Macedonia; 11 National Reference Laboratory on Vector-Borne Pathogens, Leptospira and Listeria, Microbiology Department, National Center of Infectious and Parasitic Diseases, Sofia, Bulgaria; 12 Faculty of Public and One Health, University of Thessaly, Karditsa, Greece; 13 Epidemiology and Biostatistics Task Force, Balkan Association for Vector-Borne Diseases, Novi Sad, Serbia; 14 National Laboratory of Virology, Szentágothai Research Centre, University of Pécs, Pécs, Hungary; 15 School of Biomedical Sciences, University of Plymouth, Plymouth, United Kingdom; 16 Institute of Biology, Faculty of Sciences, University of Pécs, Pécs, Hungary

**Keywords:** Crimean-Congo haemorrhagic fever, Outbreak, Tick-borne diseases, Public health surveillance, One Health approach

## Abstract

**Background:**

Crimean–Congo haemorrhagic fever (CCHF) is a severe illness characterised by fever, bleeding and high case-fatality rates. The disease is caused by CCHF virus (CCHFV), transmitted by ticks and infectious body fluids and tissues.

**Aim:**

After CCHF was diagnosed in three persons in 2023, we aimed to investigate the presence of antibodies against CCHFV in healthcare workers (HCW), sheep and goats, and of CCHFV in ticks, in an area in North Macedonia and characterise virus strains.

**Methods:**

In 2023, we collected blood samples from HCWs involved in treating CCHF patients and sera and ticks from sheep and goats in the village in North Macedonia where the index case resided. The blood samples were analysed by ELISA. Ticks were tested for presence of CCHFV, and the virus from a CCHF case was sequenced.

**Results:**

Samples from four of 52 HCWs and 10 of 17 small ruminants had antibodies against CCHFV. The virus was not detected from any of the 24 *Rhipicephalus bursa* ticks. The virus strain from the index case clustered with regional strains within the Europe-1 lineage (genotype V) group and was closest to strains from Kosovo^‡^.

**Conclusion:**

This report shows CCHFV is endemic in North Macedonia. Raising awareness of the risk factors and educating people about the measures they can take to reduce exposure to the virus is important. Healthcare workers need to be aware of the disease. Early detection, robust diagnostic methods, surveillance and collaborative efforts are necessary to prevent and control CCHF in the affected regions.

**Key public health message**

**What did you want to address in this study and why?**

Crimean–Congo haemorrhagic fever virus (CCHFV) can cause severe disease with symptoms of fever, vomiting and bleeding. The virus is transmitted by tick bites or contact with infected blood or tissue from animals or humans. In 2023, CCHFV was detected in three persons in North Macedonia. We wanted to identify potential reservoirs and high-risk groups to help inform public health measures for preventing and controlling future CCHF outbreaks.

**What have we learnt from this study?**

Of the 52 healthcare workers, four had been exposed to the virus, and 10 of the 17 tested sheep and goats. We did not detect the virus from the 24 ticks collected from the sheep and goats. The virus from a diseased person was similar to previously detected viruses from Kosovo^‡^. In conclusion, CCHFV is circulating in North Macedonia.

**What are the implications of your findings for public health?**

Our findings suggest that further CCHF cases can be expected and that there might have been CCHF cases previously not diagnosed. Information on how to prevent tick bites and transmission from diseased persons to healthcare workers is necessary. Healthcare workers need to be aware of the disease and test suspected cases. Robust diagnostic methods and surveillance of the virus are important.

## Introduction

Crimean–Congo haemorrhagic fever (CCHF) is a severe illness caused by CCHF virus (CCHFV; *Orthonairovirus haemorrhagiae*), which is usually transmitted by ticks but can also be transmitted via contact with infected animals or human blood or tissues. The illness is characterised by fever, bleeding and high mortality [1]. The disease has been reported across a large geographic area, including Africa, the Middle East, Asia and parts of southern and southeastern Europe [2]. The World Health Organization (WHO) has included CCHF among the priority infectious diseases with pandemic potential [3].

During the last decades, CCHF outbreaks in humans have been reported in eastern and south-eastern Europe, especially in Albania [4], Kosovo^‡^ [5] and Bulgaria [6]. Alongside human cases, there have been reports of seropositivity among wild animals and humans in the region, extending as far north to Hungary [7-9] and Romania [10] Although several tick species are capable to act as vectors for CCHFV, ticks of the genus *Hyalomma* are the primary vectors of concern since they are highly adapted for virus maintenance and transmission of multiple CCHFV genotypes, acting as both a vector and a reservoir [2,11]. As *Hyalomma* ticks are emerging and spreading in Europe, there is a high risk of CCHF emergence and re-emergence across the continent, influenced by multiple factors such as climate or human behaviour [12,13].

In addition to ticks, CCHFV can also be transmitted from a patient to another person by direct contact with blood or body fluids. Thus, healthcare workers (HCWs) taking care of patients with CCHF are at risk of virus exposure. Since there are currently no licensed vaccines or a specific antiviral treatment, protective clothing to prevent tick bites and stringent biosecurity measures among HCWs are the best ways to prevent transmission. Increasing awareness of the risk and continuous surveillance are essential to reduce the risk of human infections [14].

In the summer of 2023, CCHF was diagnosed in three persons in North Macedonia. The index case died, and one case was a HCW taking care of the index case [15].

The documentation of this recent cluster in North Macedonia (July–August 2023), along with genomic data on CCHFV, is important as the previous reported cases of CCHF in North Macedonia occurred in 1970 [16]. Furthermore, re-emergence of CCHF in North Macedonia warrants cross-sectoral assessment under a One Health approach, with integration of entomological, veterinary and clinical data to gain insight into the exposure to CCHFV in the affected area in North Macedonia where the index case resided and HCWs were treating CCHF cases. The nearest cases to the North Macedonian cluster were reported in Bulgaria [17], Greece [18], Kosovo [5] and Albania [19].

We aimed to extend the investigation of this CCHF cluster in North Macedonia, by detailing its epidemiology, laboratory investigations and control measures, while emphasising the importance of surveillance, prevention and international collaboration in preventing and controlling emerging infectious diseases.

## Methods

**Figure 1:**
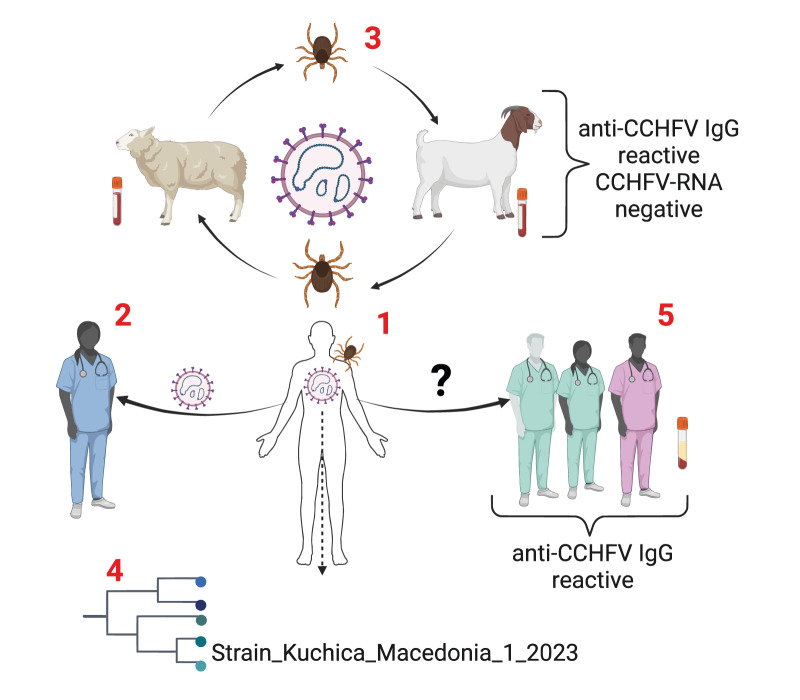
Description of the main activities during and after a cluster of Crimean–Congo haemorrhagic fever, North Macedonia, 2023

CCHF: Crimean–Congo haemorrhagic fever; CCHFV: Crimean–Congo haemorrhagic fever virus.

The most important activities during and after the detection of the 2023 CCHF cluster in North Macedonia are presented in Figure 1.

1. The index case, presenting with fever, rash, musculoskeletal pain, headache and vomiting was initially hospitalised for suspected rickettsiosis [15].

2. Case 2, a healthcare worker treating the index case [15].

3. An epidemiological study was conducted in the affected area in North Macedonia where the index case resided. Ticks and blood from farm animals (sheep and goats) were collected. The ticks were identified morphologically and analysed for detection of CCHFV with real-time quantitative reverse transcription PCR (RT-qPCR). Blood samples from sheep and goats were screened for CCHFV antibodies with ELISA.

4. Sequencing and phylogenetic analysis of the CHHFV from the index case.

5. After the outbreak was contained, antibodies against CCHFV were analysed with ELISA in sera from healthcare workers of the Clinic for Infectious Diseases in Skopje (CIDS).

Figure created with BioRender (https://www.biorender.com/).

### Sampling of humans

Blood samples from the index patient and Patient 2 were taken as described previously [15]. From Patient 3, 2 mL of blood was collected on 12 August 2023 (day 5 after symptom onset) via venepuncture in BD Vacutainer spray-coated K2EDTA tubes (BD, Oakville, the United States (US)). The blood samples were stored at −80°C until further analysis.

In November 2023, we took blood samples from 52 HCWs of the Clinic for Infectious Diseases in Skopje: HCWs with direct patient contact or contact with patient blood (n = 42) and non-medical staff (n = 10). Upon signing an informed consent, basic demographic information (i.e. sex and age) was collected through face-to-face interviews immediately prior to venepuncture.

### Sampling of farm animals

On 10 August 2023, we visited three farms (one sheep farm, one goat farm and one farm with sheep and goats) in the affected area in North Macedonia where the index case had resided. On these farms, we visually inspected 17 animals (8 sheep and 9 goats) for the presence of ticks, focusing on areas where ticks are predisposed to attach, such as the udders, external genitalia, inner thighs, perineum, base of the tail, ears and the regions surrounding the eyes.

We collected ticks using fine-tipped forceps and placed them in individually labelled vials and transported to the Department of Parasitology and Parasitic Diseases of the Faculty of Veterinary Medicine-Skopje in a cool box (4–8°C). In addition, we took blood samples from the same animals.

### Detection of Crimean–Congo haemorrhagic fever virus

Blood samples from Patients 1–3 were tested for CCHFV using the Viasure Crimean-Congo hemorrhagic Fever Virus Real Time PCR Detection Kit (Certest, Zaragoza, Spain). Ticks were identified morphologically [20], pooled into groups of six ticks and processed as described by Badji et al. [21]. For quantification of CCHFV, we applied real-time quantitative reverse transcription PCR (RT-qPCR) protocol on previously collected blood samples (n = 3), as described by Sas et al. [22]. From tick samples, RNA was extracted using SaMag 24 (Sacace Biotechnologies, Como, Italy) automatic nucleic acid extractor, while RNA from blood samples was extracted using Direct-zol RNA Kit (Zymo Research, US).

### Antibodies against Crimean–Congo haemorrhagic fever virus

Blood samples from the HCWs were tested for IgG antibodies against CCHFV using a recombinant ELISA kit VectoCrimean-CHF-IgG (VectorBEST, Novosibirsk, Russia). The assay is based on the nucleoprotein antigen (rNP) of CCHFV and includes internal positive and negative controls. The test results were interpreted according to the manufacturer’s instructions.

Blood samples from the animals were tested for CCHFV antibodies using ID Screen CCHF Double Antigen Multi-species ELISA kit (Innovative Diagnostics, Grabels, France), following the manufacturer's protocol. The optical densities (OD) were read at 450 nm using the Multiskan FC Microplate Photometer (Thermo Fisher Scientific, Waltham, US).

### Characterisation of Crimean–Congo haemorrhagic virus

#### Nucleic acid extraction and real-time PCR

The nucleic acid extraction was performed in the biosafety level (BSL) 4 laboratory of the National Laboratory of Virology, Pécs, Hungary. We used 200 μL of whole blood sample for the extraction with the Direct-zol RNA Kit.

We used Crimean–Congo haemorrhagic fever virus specific primers and probes of Atkinson et al. [23]. For the quantification, we used the Luna Universal One-Step RT-qPCR Kit (New England Biolabs, Ipswich, US) and for cycling, we used the Mic qPCR platform (Bio Molecular Systems, Upper Coomera, Australia). Cycling conditions were as follows: 55°C for 11 min, 95°C for 1 min, followed by 40 cycles of 95°C for 10 s, 55°C for 60 s and 72°C for 20 s. Patient samples, in which CCHFV RNA was detected, were subjected to further sequencing.

#### Sequencing

Following a viral enrichment protocol on 200 μL whole blood sample, using filtering and enzymatic digestion [24], the nucleic acid isolation was performed with Zymo Direct-zol-96 RNA Kit (Zymo Research). The RNA library was generated using NEBNext Ultra II Directional RNA Library Prep for Illumina (New England Biolabs). Briefly, 10 ng of the total RNA was used as input for fragmentation step and complementary DNA (cDNA) generation was performed using random primers. Thereafter, the cDNA was end-prepped and adapter-ligated, then the library was amplified according to the manufacturer’s instructions. The quality of the libraries was checked on 4200 TapeStation System using D1000 ScreenTape (Agilent Technologies, Santa Clara, US), the quantity was measured on Qubit 3.0 (Thermo Fisher Scientific). Illumina sequencing was performed on the NovaSeq 6000 instrument (Illumina, San Diego, US) with 2 × 151 run configuration. Raw reads were quality controlled with FastQC version 0.12.1 and error corrected and quality trimmed with NanoFilt version 2.8.0 (https://github.com/wdecoster/nanofilt). The genomes and genome parts were de novo assembled with SPAdes version 3.15.5 (https://github.com/ablab/spades) (raw reads as SPAdes has a built-in error correction and quality trimming function) and MEGAHIT version 1.2.9 (https://github.com/voutcn/megahit) (corrected reads) and mapped to the closest match in GenBank (https://www.ncbi.nlm.nih.gov/genbank/) using Geneious Prime (https://www.geneious.com/) version 2023.1.1. Illumina reads were mapped to the consensus sequences from the former step and further corrected in Geneious Prime. For multiple sequence alignments, sequence and phylogenetic analyses Geneious Prime 2023.1.1 and PhyML software (https://github.com/stephaneguindon/phyml) version 3.0 were used.

#### Phylogenetic analyses

We performed a separate phylogenetic analysis for the complete coding sequence of the three viral segments. The trees were constructed with the Geneious Tree Builder feature implemented in Geneious Prime version 2023.2.1 software. During the analyses, we used the neighbour-joining tree build method with Tamura-Nej model [25] with the Bootstrap resampling method option with 1,000 replicates. The constructed trees were visualised and edited in iTOL online tool [26].

## Results

### Detection of Crimean–Congo haemorrhagic fever virus

During routine diagnostic process in Skopje, viral RNA via commercial PCR was detected in blood of all three cases [27,28]. When the same blood samples were tested retrospectively via RT-qPCR analysis, CCHFV RNA was detected only in the sample of the index case. We found 24 ticks from seven sheep (2–5 ticks per animal) on one sheep farm. No ticks were seen on the other two farms, but the animals had been treated with ivermectin 1 day before the veterinary visit. The collected ticks were non-engorged *Rhipicephalus bursa* nymphs (n = 9) and female adults (n = 15). We did not detect CCHFV from the tick pools.

### Antibodies against Crimean–Congo haemorrhagic fever virus

Anti-CCHFV IgG antibodies were detected from samples of four of 52 HCWs (Table). Notably, none of the HCWs had a previous history of illnesses with symptoms compatible with haemorrhagic fever. One of the seropositive HCWs was a medical nurse who had been caring for the index patient.

**Table:** Serological response against Crimean–Congo haemorrhagic fever virus in healthcare workers, North Macedonia, November 2023 (n = 52)

Occupation	Male (n = 19)		Female (n = 33)		Total
	Positive	Negative	Positive	Negative	
HCW with direct patient contact or contact with patient blood	1	14	2	25	42
Non-medical staff	1	3	0	6	10

HCW: healthcare worker.

**Figure 2:**
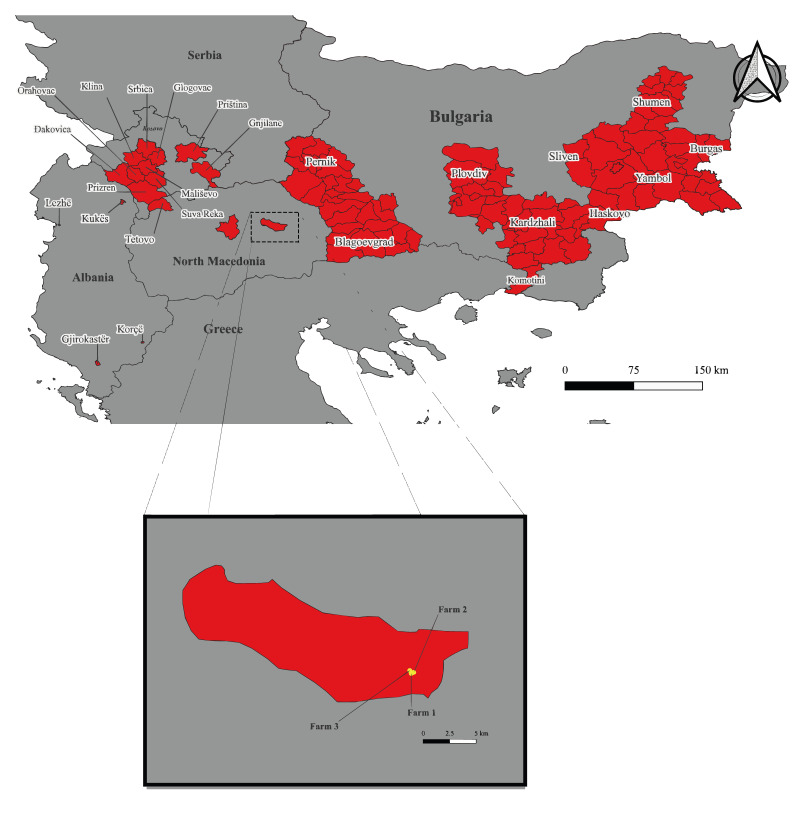
Location of small ruminant farms tested for Crimean–Congo haemorrhagic fever virus, 2023 (n = 3) and human cases of Crimean–Congo haemorrhagic fever 2001–2023 (n = 239), North Macedonia and neighbouring countries^a^

^a^ Albania, Bulgaria, Greece, Kosovo^‡^, Serbia.

We detected anti-CCHFV antibodies from animals (10/17) on all three farms (Figure 2). On the farm with sheep and goats, all five animals had antibodies, four of five animals on the sheep farm and one animal on the goat farm had antibodies.

Municipalities with reported human cases of Crimean–Congo haemorrhagic fever are marked in red [4,19,32,46-54].

Farms in in the affected area in North Macedonia where the index case resided, and farm animals were tested for antibodies against Crimean–Congo haemorrhagic fever virus and ticks for Crimean–Congo haemorrhagic fever virus are marked in yellow.

A shapefile of countries for mapping at district and municipality levels is available in the GADM database of Global Administrative Areas (version 3.6, April 2020, https://gadm.org/). The map was generated by using QGIS version 3.12 (QGIS Development Team, 2020).

### Characterisation of Crimean–Congo haemorrhagic fever virus

We were able to detect viral RNA via RT-qPCR only from the sample of the index patient. After sequencing on Illumina platform, we obtained the whole coding sequence of all three CCHFV genome segments from the blood sample (coverage: 54.0 ± 17.6 × (segment S), 86.0 ± 84.2 × (segment M) and 65.5 ± 43.2 × (segment L); mapped reads: 596 (segment S), 3,035 (segment M) and 5,290 (segment L)). Sequences of segments S, M and L have been deposited in the GenBank database under accession numbers PP729064, PP729065 and PP729066, respectively.

**Figure 3:**
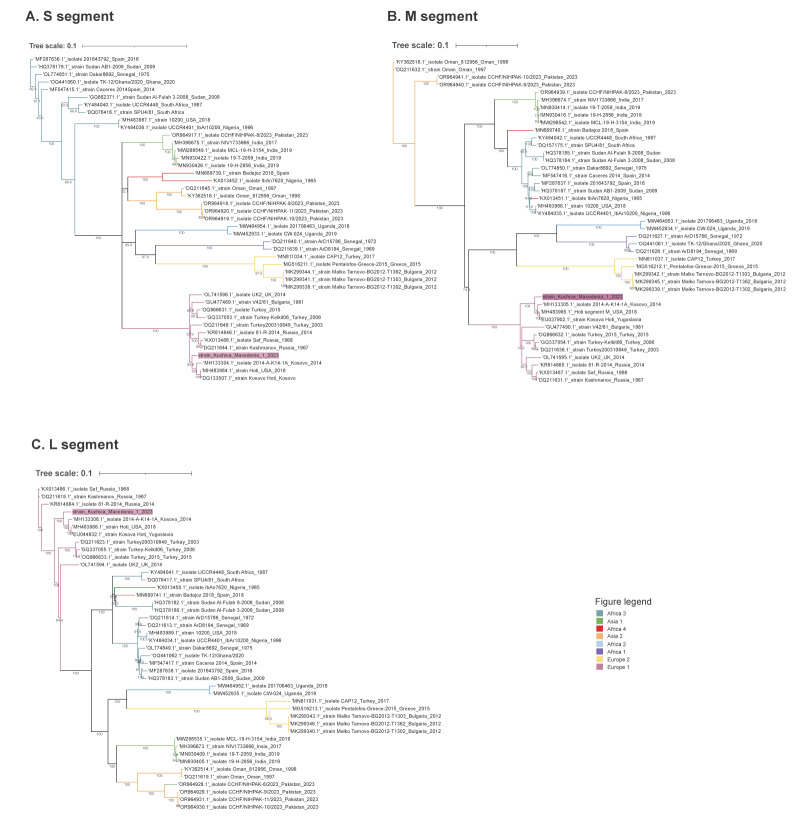
Neighbour-joining phylogenetic trees of three viral genomic segments of Crimean–Congo haemorrhagic fever virus from one patient, North Macedonia, 2023 and sequences from Genbank (n = 3)

Based on the phylogenetic analysis, the strain clustered with regional strains within the Europe-1 lineage (genotype V) group. The homology (98.58% at the amino acid level for M segment) and phylogenetic position (clustering with Kosovo Hoti strains) confirmed the similarity to the CCHFV Hoti strain. The diversity of CCHFV in Kosovo was previously reported with a maximum of 1.9% difference at the amino acid level homology of the M segment [29], and our sequences are within this range. However, we observed a slightly different position from the Kosovo cluster as this novel sequence is positioned on a separate node. Figure 3 shows the phylogenetic position of our sequence data.

The sequence of this study is highlighted with coloured background. Sequences from GenBank (PP729064–PP729066).

## Discussion

The CCHF cluster in 2023 is the first one in North Macedonia since the summer of 1970 when 13 individuals diseased in Chiflik (Zhelino municipality) and two died [16]. The current cluster occurred in regions in eastern Macedonia ca 70 and 100 km from the location of the 1970 outbreak. Although *Hyalomma* ticks are known to have been present in North Macedonia for more than a century [30], there are no continuous CCHFV monitoring programmes (i.e. tick analysis and serosurvey in sentinel farm animals or individuals at-risk). Until this recent cluster, information on the CCHFV circulation in North Macedonia was scarce [31], although the country is neighbouring the CCHF endemic and hyperendemic regions of Kosovo [5] and Bulgaria, where all CCHFV isolated from humans so far belong to genotype V (Europe 1) [32,33].

We demonstrated serological response in small ruminants in the affected area in North Macedonia where the index case resided, as has been seen in sheep in the endemic regions in Bulgaria [34]. Sheep are able to sufficiently replicate CCHFV and can be considered as sentinels for monitoring of CCHFV circulation [35,36]. As we tested a small number of samples and did not use another serological test, we cannot define the role of sheep and goats in CCHFV maintenance and transmission. Therefore, further studies are warranted to identify the frequency of CCHFV exposure in small ruminants and to determine if other farm animals (e.g. cattle, horses) can be used as sentinels for CCHFV circulation.

The phylogenetic analysis revealed that the CCHFV from the index case was close (i.e. positioned on the same phylogenetic clade) to the strains from the nearest known CCHFV hotspot in Kosovo. Although we only sequenced one strain, we consider the CCHF outbreak was caused by an endemic strain rather than by a newly introduced virus from neighbouring countries endemic for CCHF [4,17,18].

Crimean–Congo haemorrhagic fever may be underdiagnosed in the North Macedonian population as the infection can be asymptomatic or present with mild symptoms [37]. Of the 52 tested HCWs, four had antibodies against CCHFV, suggesting previous virus exposure. Similar and higher proportions of seropositivity have been noted in neighbouring Bulgaria [6] and associated with wide distribution of low pathogenic CCHFV lineage Europe 2 in addition to the high pathogenic lineage Europe 1 [38]. Since 2009, CCHF has been detected in new areas in Bulgaria close to the border of North Macedonia [39]. Taken together, all these findings show that CCHFV is endemic in North Macedonia and further CCHF cases might be expected.

These events led to the establishment of a new association dedicated to vector-borne diseases on the Balkan Peninsula – Balkan Association for Vector-Borne Diseases (https://www.bavbd.org/), which held its first assembly in December 2023. During this inaugural assembly, plans were formulated to enhance future international collaboration and strengthen national diagnostic and treatment capacities, while also implementing prevention strategies.

As subclinical infections of CCHFV may be more common than appreciated [1] and a continuous surveillance of CCHFV virus in North Macedonia is lacking, it is difficult to perform a comprehensive risk assessment. The current CCHF surveillance in North Macedonia does not allow us to assess the prevalence of CCHFV in the population of the affected areas where CCHF cases have emerged. In this study, we relied on a single serological assay for testing both humans and animals. Although the assay is internally validated, no additional serological methods were employed for confirmation. Prevalence studies in humans and animals should be prioritised alongside the collection of environmental data. Factors affecting the presence of an autochthonous strain of CCHFV in North Macedonia and its transmission are not fully elucidated. Key contributors likely include climate change affecting tick ecology and virus incubation in vectors, as well as movement of animals and the spread of ticks through livestock trade and wildlife migration, all of which affect the transmission dynamics. As the annual average temperatures in North Macedonia are increasing [40], repercussion on tick activity and host parasitic load is expected [41,42], thus promoting CCHFV circulation and increasing the chance for susceptible individuals to be exposed to a bite of an infected tick.

Our study highlights the importance of clinical vigilance in the region and calls for international action to fully understand the regional risk of CCHFV infection and uncover the natural transmission patterns of the virus in the whole Balkan region. The recent cluster underlines the necessity of increasing awareness among medical professionals by conducting nationwide training programmes focused on early diagnosis, case management and preventive measures. Increasing public awareness in the country through targeted educational campaigns and dissemination of information are crucial, with primary care physicians and public health authorities serving as the frontline in educating communities about preventive measures. Ensuring nationwide access to robust diagnostic techniques and strengthening diagnostic capacities is also critical for timely detection and response to future outbreaks.

Building this groundwork is essential for establishing sustainable surveillance systems which should incorporate monitoring of vectors and potential sentinel populations. Additionally, using simple early warning tools, such as the Epidemic Volatility Index (EVI) can provide early warnings and help in the timely mobilisation of resources to areas at risk, thereby preventing further transmission [43,44]. By analysing real-time data, outbreaks can be predicted and monitored, which is especially crucial for diseases like CCHF, given its potential to escalate rapidly and lead to high fatality rates in certain regions [45], although outbreaks in Europe have so far been limited in scale and impact. Incorporating advanced predictive tools, including Bayesian predictive values and other surveillance technologies, into the surveillance systems not only enhances our response capabilities but also significantly mitigates the impact on public health in North Macedonia and other CCHF-endemic states.

## Conclusion

In conclusion, implementing a comprehensive One Health surveillance programme is essential for monitoring CCHFV circulation, which should include regular tick collection, serosurveys in sentinel animals and systematic data collection on environmental factors. Increasing awareness on CCHF among healthcare workers and public is essential to improve diagnosis and prevention of cases. Strengthening national diagnostic capacities and fostering international collaboration will enhance early detection, facilitate timely interventions and improve public health responses to CCHF outbreaks.

## ‡Note

This designation is without prejudice to positions on status and is in line with United Nations Security Council Resolution 1244/99 and the International Court of Justice Opinion on the Kosovo Declaration of Independence.
